# Editorial: Exercise to enhance mental health

**DOI:** 10.3389/fnhum.2022.1082218

**Published:** 2022-11-24

**Authors:** Wendy A. Suzuki

**Affiliations:** Center for Neural Science and Psychology, New York University, New York, NY, United States

**Keywords:** exercise, affect, cognition, aging, inhibitory control

The articles herein offer a snapshot of recent research in the area of exercise's impact on affect and cognition. Addressing both well-studied populations as well as novel populations, these seven articles add to our understanding by looking at how physical exercise can affect cognitive decline and dementia; mood symptoms related to exposure to trauma; psychosocial functioning; stress-related symptoms; large-scale brain networks; and mood and body image data in moderately fit individuals. Two articles examine the potential synergistic effects of combining exercise with mindfulness training, and another reported on a study of the potential impact of acute exercise on impulse control and error detection.

Probably the most well-established beneficial effect of exercise on the brain has been finding showing broad improvement to cognition and affect in older adults. Babaei and Azari provide a review of this research, showing the positive impact of exercise on brain functioning by investigating whether exercise has the capacity to offset and perhaps delay cognitive decline and dementia associated with aging. They summarize the positive effects of exercise on memory performance, including working memory, as well as executive function. Looking at the possible mechanisms underlying improvements in memory, the authors cite research pointing to the relationship between regular physical activity's ability to improve brain circulation; neurogenesis; the elevation of certain neurotrophic factors, such as BDNF, IGF-1, and irisin, which all involve hippocampal plasticity and long term memory. Additional mechanisms such as mitochondrial biogenesis and the release of numerous signaling molecules, including myokines and adipokines, may also explain the improvements in neurocognitive performance for older adults. In a somewhat surprising result Duzel et al. describe no evidence for improved psychosocial functioning in older adults after a 6-month physical exercise intervention. This stands in contrast to one study that showed a positive correlation but focused on participants with MDD, many of whom were on anti-depressant medication, pointing to the nuance of the Duzel et al. study relative to the wider literature that shows exercise's positive effect on mood and social engagement.

Two articles address the well-studied observation that exercise (with or without meditation) has a beneficial effect on mood. Demmin et al. addressing a pressing practical issue, examine the combination of meditation and aerobic exercise on mental health and wellbeing in teaching during the COVID-19 pandemic. Given the substantial and ongoing research into the beneficial effects of mindfulness activities on wellbeing and cognition, especially as it relates to children's academic performance, the authors shifted their focus to teachers, given the long-established evidence that teaching is a high stress job. The pilot study led a cohort of teachers through an intervention of MAP training (a combination of 30 min focused-attention and slow-walking meditation, both done in silence), followed by 30 min of aerobic exercise. Despite the relatively small sample size, the study results suggest that the combination of meditation training and aerobic exercise “may prevent or at least mitigate some of the mental health symptoms that arose during the height of the COVID-19 pandemic,” pointing to future applications of the dual-modality approach to building resilience. Basso et al. explore both affect and cognitive function in a less studied population, namely, moderately fit middle-aged adults, to determine if clear cognitive, mood, and motivational benefits can be seen in already fit individuals. This study reported significant associations between the number of workouts and a wide range of mood, exercise, motivational, and cognitive tasks, including hippocampal-dependent spatial navigation. Taken together, these studies provide more support for the well-studied positive effects of exercise in older adults as well as affirm both the practical applications of the positive effects of exercise on mood in hard-working (and very likely stressed out) teachers as well as in moderately fit adults.

Shaw et al. provide new insights on the mechanisms that underlie the combined effects of physical activity and neurofeedback as measured by EEG and neuroimaging. This study builds on well-established research demonstrating the positive effects on mood of both aerobic exercise and mindfulness activities. To further understand the neural mechanisms of such mood improvement, the investigators studied the discrete effects of aerobic exercise and mindfulness-like neurofeedback training on intrinsic network synchrony (ICN), including how the default mode network (DMN), the central executive network (CEN), and the salience network (SN) overlap in function and communicate with one another at the cellular level. The 8-week intervention used EEG-based neuroimaging scans before and after to measure task-related skills in an effort to further understand the synergistic impact of combining lifestyle interventions such as mindfulness training and aerobic exercise on cognitive functioning and mood.

Like the “real world” effects of exercise on teachers during COVID, the final two articles in this volume explore the effects of aerobic exercise in two different (sub)clinical/pathological conditions. Mizzi et al. explore the impact of aerobic exercise on mood symptoms in trauma-explosed young adults while Yu et al. explore the effect of an acute exercise intervention on both inhibitory control and error detection in violent male perpetrators. In their pilot study, Mizzi et al. examine the effects of physical exercise on young adults suffering from sub-clinical symptoms of PTSD, including anxiety and depression. Given the high incidence of trauma in the Canadian population, they led 25 “low active” young adults through an 8-week exercise intervention, comparing them to a control group. Despite the small sample size, the authors conclude that the intervention significantly decreased negative mood symptoms and improved emotional regulation compared to the control group. Similarly Yu et al. report positive effects of exercise on inhibitory control in violent male criminals. Given that inhibitory control is a core component of executive function (EF), the results of this study point to possible interventions for executive dysfunction in the wider population.

The articles in this volume expand our understanding of the effects of exercise in both older and younger populations and highlight the synergistic effects of combining exercise and meditation/feedback. As a whole, the articles represent an expansion of exercise effects beyond depression and anxiety to violent criminals, a group who present extreme deficits in impulse control and error detection, two features of cognitive-affective processing.

## Author contributions

The author confirms being the sole contributor of this work and has approved it for publication.

## Conflict of interest

The author declares that the research was conducted in the absence of any commercial or financial relationships that could be construed as a potential conflict of interest.

## Publisher's note

All claims expressed in this article are solely those of the authors and do not necessarily represent those of their affiliated organizations, or those of the publisher, the editors and the reviewers. Any product that may be evaluated in this article, or claim that may be made by its manufacturer, is not guaranteed or endorsed by the publisher.

